# Development and Characterization of Biodegradable Films on Native and Esterified Peruvian Purple Yam (*Dioscorea trifida*) Starches and Tara Gum

**DOI:** 10.3390/polym17202754

**Published:** 2025-10-15

**Authors:** Paola Cornejo, Naomi Chalco, Sebastian Gutiérrez, Katherine Junco, Ronal Lopinta, Fiorela Peña-Carrasco, Carmen Velezmoro-Sánchez, Patricia Martínez-Tapia

**Affiliations:** 1 Círculo de Investigación de Biopolímeros y Nanomateriales, Facultad de Industrias Alimentarias, Universidad Nacional Agraria La Molina, Lima C.P. 15024, Peru; 2 Programa de Doctorado en Nutrición, Universidad Nacional Agraria La Molina, Lima C.P. 15024, Peru; 3 Departamento de Ingeniería de Alimentos, Facultad de Industrias Alimentarias, Universidad Nacional Agraria La Molina, Lima C.P. 15024, Peru

**Keywords:** purple yam, esterified starch, biodegradable films, water permeability, disintegration

## Abstract

The aim was to evaluate if purple yam starch esterification with octenyl succinic anhydride (PYS-OSA) enhances the properties of purple yam starch (PYS)-based films in a blend with tara gum (TG). PYS was isolated from purple yam tubers (PYTs) with distilled water; then, starch was dual-modified by ultrasound (as a pretreatment) and esterification (PYS-OSA). The films PYS:TG and PYS-OSA:TG were characterized through physicochemical and mechanical characterization. The thermal properties (*T_o_*, *T_c_*, *T_p_*, and Δ*H*) of PYS-OSA decreased in the range of 3.4–7.6% compared to PYS. Fourier transform infrared spectroscopy (FTIR) confirmed esterification, revealing two new absorption bands at 1563.0 and 1726.5 cm^−1^, and the degree of substitution (DS) was 0.023. The moisture content and solubility in water were 50.7 and 40.5% greater, respectively, for PYS-OSA:TG films compared to PYS:TG ones, but both films exhibited similar optical properties. The tensile strengths of PYS-OSA:TG films were higher than those of PYS:TG ones; however, the elongation at break was lower. PYS:TG and PYS-OSA:TG films were disintegrated by more than 70% after 13 days of being buried in soil. This work contributes to a better understanding of the starch isolated from purple yam tuber, with potential relevance for sustainable packaging applications.

## 1. Introduction

Petroleum-derived plastics pollute the environment due to their degradation taking a long time, so the alternative, in order to enhance the environment, is to use biopolymers [1,2,3]. In different industries, natural biopolymers (such as starch, gums, proteins, and chitosan, among others) are being studied as a matrix of biodegradable or edible films because they are abundant, biodegradable, cheap, biocompatible, non-toxic, and renewable resources [1,4,5]. Regarding the food industry, this type of film is an alternative form of sustainable packaging to cover and extend the shelf-life of food products [4].

Purple yam (*Dioscorea trifida*) is a well-known crop in parts of Brazil, Colombia, Peru, and other countries in South America [6,7]; however, this crop is still considered an “orphan” and is underutilized because it is mainly planted by small-scale farmers using traditional agricultural methods [7]. In Peru, the popular name of purple yam is purple *sachapapa*, which has been found in some publications [6,7,8,9], and it is cultivated mainly in the Peruvian rainforest and in the Loreto, Madre de Dios, San Martín, and Ucayali regions [10]. Yam tubers are rich in carbohydrates, mainly starch [10]; their starch has some interesting functional properties, similar to cereals [4]. On the one hand, yam starches from different species have been characterized by several researchers [4,11,12,13]. On the other hand, yam-starch-based films have shown better mechanical properties than cassava starch [14], and applying these films on apples maintained the fruit quality [4].

Tara gum (TG) is also a potential edible-film-forming candidate for diverse applications in the food packaging industry [15]. It is obtained from the seeds of the Peruvian tara tree (*Caesalpinia spinosa*), which has been widely used in food and industrial applications as a thickener and stabilizer [1,16]. TG is a galactomannan that consists of a linear main chain of (1–4)-β-D-mannopyranose units linked with branched chains of (1–6)-α-D-galactopyranose (mannose/galactose = 3) and presents lower galactose substitution, which means it can produce a stronger film [16]. TG has been used to produce UV-irradiated nanocomposite tunta starch/tara gum (TS:TG)-based-films, which showed high stiffness and strength as well as less elongation. Additionally, these nanocomposite films were almost completely degraded after 5 days of burial [1].

Considering the good properties of purple yam starch (PYS) and TG, combining these biopolymers might produce a good blend film for use in packaging. Thus, this work aimed to evaluate the properties of the blending of PYS:TG and PYS-OSA:TG films. The films were characterized by their thermal stability, rheological behavior, optical and mechanical properties, and water vapor permeability. This study hypothesizes that the esterified PYS (PYS-OSA) could enhance these biodegradable films’ physicochemical, optical, mechanical, and disintegration properties. To our knowledge, current studies concerning the development of biodegradable films based on esterified PYS are scarce.

## 2. Materials and Methods

### 2.1. Materials

**Figure 1:**
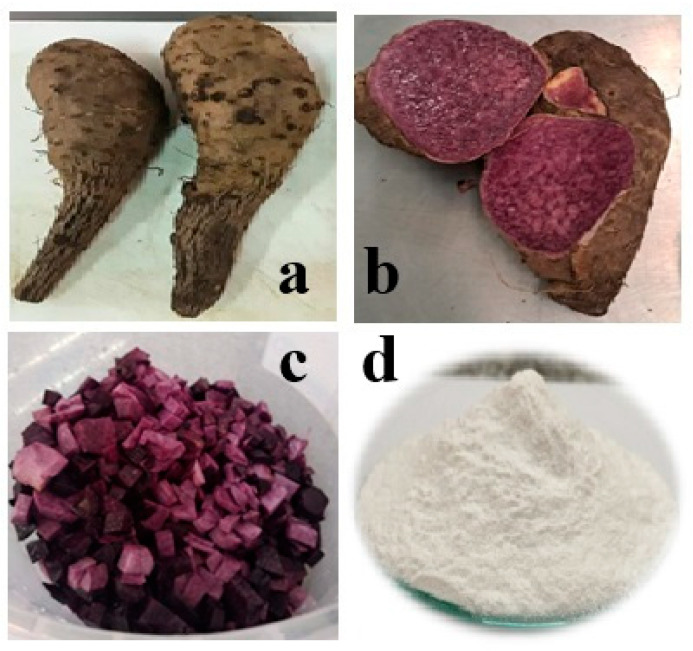
Peruvian purple yam (*Dioscorea trifida*) tubers: (**a**) entire tuber, (**b**) half pieces, (**c**) small cubes, and (**d**) isolated starch from yam tubers.

Peruvian purple yam (*Dioscorea trifida*) tubers were purchased from a local farmer in Tingo María, Huánuco, Peru (latitude 9°18′05″ S, altitude 76°02′10″ W). Photos of the entire tubers and their half pieces are shown in Figure 1a and Figure 1b, respectively. The 2-octen-1-ylsuccinic anhydride (OSA), glycerol, and other reagents were purchased from Sigma-Aldrich Chemical Co. (Saint Louis, MO, USA). Tara gum (TG) (viscosity at 20 °C: ~6000 mPa·s) [1] and sodium bromide (NaBr) were purchased from PoliFood Perú S.A.C. and Movilab del Perú S.A.C. (Lima, Peru), respectively. Other chemical reagents used in this research were of analytical grade.

### 2.2. Proximate Analysis of Peruvian Purple Yam Tubers

The methods used to examine purple yam tubers were moisture content [17], total protein [18], fat [19], ash [20], and crude fiber [21]. The nitrogen-free extract content was determined as the remainder after subtracting the total percentage of other components from 100. The phosphorus content was measured by AOAC’s method (986.24) [22]. All analyses were performed in triplicate.

### 2.3. Starch Extraction

Starch (PYS) from Peruvian purple yam tubers was extracted using a slight modification of the method described by Martínez et al. [23]. Purple yam tubers were washed, peeled manually, and cut into small cubes (Figure 1c). A sample of 3 kg was ground in a blender with 3 L of distilled water. The juice was filtered through a sieve, and the residue remaining on the sieve was washed with distilled water. The filtrate was collected in a vessel and left undisturbed for four hours, and the residue on the sieve was discarded. The starch cake settled in the vessel, the supernatant was decanted, and then the cake was again suspended with two volumes of distilled water and left at rest for sedimentation. This procedure was repeated four times. The starch cake was dried at 40 °C in an air oven (Venticell 55, MMM, Munich, Germany) for 24 h. Dried starch was ground gently in a lab grinder, sieved, and packed in airtight jars till analysis (Figure 1d).

### 2.4. Starch Modification (Esterification with OSA)

Purple yam starch (PYS) was ultrasound-pretreated and esterified with OSA according to the method proposed by Martínez et al. [24]. Briefly, starch slurries of 30% (*w*/*w*, dry weight) were stirred for 15 min and treated with an ultrasonic dispersion instrument (VC 505, Sonics, Dallas, TX, USA) equipped with a transductor, a generator, and a titanium sonotrode (15 mm tip diameter) for 5 min, with pulse durations of 60 s on and 10 s off (20 kHz); during sonication the slurries were immersed in an ice-water bath. After sonication, the starch slurry was made with 3% OSA reagent (five parts diluted with absolute alcohol, *v*/*v*), slowly added over 2 h (drop by drop). The reaction continued for 1 h more at 25 °C with continuous stirring (pH = 8.5–9.0). The slurries were neutralized to pH 7 using 0.1 M HCl. The esterified starches were separated by centrifugation at 4000× *g* for 10 min at 10 °C, washed three times with distilled water, and washed once with ethanol. The modified starch was dried in a forced-air oven (Venticell 55, MMM, Munich, Germany) at 40 °C for 24 h. The modification procedure was replicated in duplicate. Dried starch was powdered and labeled as PYS-OSA. The starch was stored in airtight jars for further analysis.

### 2.5. Characterization of Native and Esterified Starches

#### 2.5.1. Proximate Analysis and Apparent Amylose Content

Proximate composition analysis of native purple yam starch (PYS) was independently conducted for purple yam tubers (PYTs), but with the same protocols depicted in Section 2.2. All analyses were carried out in triplicate. The apparent amylose content for PYS and PYS-OSA was determined using the method of Hoover and Ratnayake [25].

#### 2.5.2. Degree of Substitution (*DS*)

The *DS* of PYS-OSA was determined according to the method proposed by Timgren et al. [26]. Briefly, 1.25 g (dry weight) of PYS-OSA was suspended in 12.5 mL of 0.1 M HCl with stirring for 30 min. The slurry was centrifuged at 4000× *g* for 10 min at 10 °C, washed once with 12.5 mL ethanol (90%), and washed two times with distilled water. The precipitate was dispersed in distilled water (75 mL), cooked in a boiling water bath for 10 min, and cooled to room temperature. The solution was titrated with 0.1 M NaOH until pH 8.3. A blank was titrated using the native starch for each modified one. The *DS* was calculated using Equation (1):

(1) $$DS=\frac{0.162\times \frac{\left(A\times M\right)}{W}}{1-\left(\frac{0.210\times A\times M}{W}\right)}\;$$

where *A* is the titration volume of 0.1 M NaOH solution (mL), *M* is the molarity of the NaOH solution, and *W* is the dry weight (g) of the modified starches.

#### 2.5.3. Particle Size Distribution

The particle size distribution determination of PYS and PYS-OSA was performed using a laser diffraction analyzer (Mastersizer 3000, Malvern Instruments Ltd., Malvern, UK). The samples were dispersed in distilled water (refractive index 1.330) using the equipment, and the distribution measurements were carried out according to Fraunhofer diffraction theory. The samples were analyzed in triplicate. The volume-based (*D* [4,3], Equation (2)) diameter was evaluated, which was influenced by large particles [27].

(2) $$D\left[4,3\right]=\frac{\sum_{i}n_{i}d_{i}^{4}}{\sum_{i}n_{i}d_{i}^{3}}\;$$

#### 2.5.4. Thermal Properties

The onset temperature (*T_o_*), peak temperature (*T_p_*), conclusion temperature (*T_c_*), and gelatinization enthalpy (Δ*H*) of PYS and PYS-OSA were determined using a differential scanning microcalorimeter (Multi-Cell DSC, TA Instruments, New Castle, DE, USA) according to the method reported by Martínez et al. [28]. Starch samples (20 mg, dry weight) were weighed directly into screw-cap ampoules (TA Instruments Hastelloy-C), and 60 μL of ultrapure water was added. The ampoules were placed into the microcalorimeter, equilibrated for 1 h at 25 °C, and heated from 25 °C to 115 °C at 2 °C/min. The runs were performed in duplicate. NanoAnalyze v.2.4.1 software was used to calculate the thermal properties.

#### 2.5.5. Scanning Electron Microscopy (SEM)

The microphotographs of native (PYS) and esterified (PYS-OSA) starch granules were evaluated with SEM (Axia ChemiSEM, ThermoFisher Scientific™, Waltham, MA, USA) using a magnification of 400×, 1500×, and 3000× in the low vacuum mode and with a CBS detector. The samples were attached to conductive carbon tape with double-sided glue in the microscope cells.

#### 2.5.6. Infrared Spectroscopy (FTIR)

The FT-IR spectra of PYS and PYS-OSA and their films (PYS:TG and PYS-OSA:TG) were obtained on a spectrometer (Nicolet iS10, Thermo Scientific Inc., Waltham, MA, USA) using a DTGS detector and the ATR SmGart iTX accessory. Each FT-IR spectrum was recorded in the wavenumber range of 4000–400 cm^−1^, with a resolution of 4 cm^−1^ and averaging 64 scans. The OMNIC 9.7.46 software was used to perform the analysis.

#### 2.5.7. X-Ray Diffraction (XRD) Pattern and Crystallinity

The method described by Martínez et al. [23] with slight modifications was used for XRD measurements. PYS and PYS-OSA were conditioned for 5 days in a desiccator with a saturated K_2_SO_4_ solution. An X-ray diffractometer (D8 Advance, Bruker Corporation, Billerica, MA, USA) registered the measurements under a standard Bragg–Brentano configuration, supplied with the ultra D/tex detector (40 mA and 30 kV) and monochromatic radiation CuKα (λ = 0.154060 Å). Diffractograms were registered in the range 3.8° < 2θ < 35° with a step size of 0.02° and a step count of 1 s. For crystallinity determination, the PeakFit 4.12 software was used to calculate crystalline and amorphous regions. The crystallinity (%) was determined as the ratio of the crystalline area to the total area (crystalline + amorphous), according to the method described by Steffolani et al. [29].

#### 2.5.8. Rheological Characteristics

The starch (PYS and PYS-OSA) suspensions of 4% (*w*/*w*) were cooked at 90 °C for 30 min and continuously stirred to prevent concentration during gelatinization. The starch pastes were cooled to 25 °C and immediately placed on the Peltier plate of a hybrid rheometer (HR-3 Discovery, TA Instruments, New Castle, DE, USA) for examination [24]. The steady-state rheological measurements of PYS and PYS-OSA pastes were conducted with a parallel plate configuration (40 mm diameter) and a 1 mm gap between the Peltier plate and probe. The steady shear test was performed over a 1 to 100 s^−1^ shear rate range. The flow behavior of the sample was modeled using the power law and Herschel–Bulkley models, as outlined in Equations (3) and (4).

(3) $$\tau =k\;\gamma^{n}$$

(4) $$\tau =\sigma_{0}+k\;\gamma^{n}\;$$

where *τ* represents the shear stress (Pa), *γ* denotes the shear rate (s^−1^), *n* is the flow behavior index, and *k* is the consistency index (Pa⋅s^n^). The coefficient of determination (adjusted R^2^) was used to determine the accuracy and reliability of the model fit.

The dynamic shear tests were conducted to understand the viscoelastic behavior of starch pastes. The amplitude sweep test was carried out on starch pastes to identify the linear viscoelastic region (LVE). PYS and PYS-OSA pastes were placed on the rheometer and maintained at rest for 5 min, with shear strain varying between 0.01 and 100 Pa at a constant frequency of 1 Hz. Afterward, a frequency sweep test was performed within the LVE range, covering frequencies from 0.01 to 10 Hz at 25 °C. The mechanical spectra were obtained by recording storage modulus (*G*′), loss modulus (*G*″), and loss tangent (*tan δ* = *G*″/*G*′) as a function of the frequency (*ω*).

### 2.6. Film Preparation

Starch-based films were developed using the solvent-casting procedure according to the methodology described by Pérez-Córdoba et al. [1]. The film-forming dispersion (FFD) was based on the starches (PYS and PYS-OSA) and their blends with tara gum (TG). The FFD containing a 4% *w*/*w* of a mix of each starch and TG (95:5 ratio) and glycerol (25 g/100 g polymers) (plasticizer) was prepared by dispersing starch and dissolving TG overnight in distilled water. These dispersions were mixed, then heated at 90 °C for 30 min at 600 rpm until gelatinization and then cooled to 50 °C. The obtained FFD was stirred for 3 min at 12,500 rpm using an Ultra-Turrax^®^ (T25, IKA, Staufen, Germany). Then, the FFD was degassed in a sonicator bath (DL 510 H Sonorex Digiplus, BANDELIN electronic GmbH & Co., Berlin, Germany) at 50 °C for 20 min. Finally, FFD was poured onto a polystyrene Petri plate (14 cm in diameter) and dried in a forced-air oven (MA035/5, Marconi, Piracicaba, Brazil) at 35 °C overnight. Once dried, the films were removed from the plates and conditioned at room temperature in desiccators containing NaBr saturated solution (58% relative humidity) for at least 5 days till characterization. Films based on PYS and PYS-OSA were labeled PYSF and PYS-OSAF, respectively.

### 2.7. Film Characterization

#### 2.7.1. Film Appearance and Thickness

The appearance and homogeneity of PYS:TG and PYS-OSA:TG were evaluated by qualitative visual inspection. The thickness was determined at 10 random points on each sample surface using a digital micrometer (Mitutoyo, 543–391, Kawasaki, Japan) with an accuracy of 0.001 mm [2]. The average value of these measurements was reported.

#### 2.7.2. Moisture Content (*MC*) and Solubility in Water (*SW*)

The films were cut into small disks (20 mm in diameter) to determine their moisture content (*MC*) and solubility in water (*SW*). *MC* was evaluated by mass reduction in three film disks in a forced-air oven (Venticell 55, MMM, Munich, Germany) at 105 °C for 24 h. For *SW* measurement, three disks were weighed (m_0_) and immersed in distilled water (50 mL) under stirring in an orbital shaker (TOU-120, MRC, Holon, Israel) at 25 °C and 80 rpm for 24 h. Film samples were removed from the solution, dried in a forced-air oven (Venticell 55, MMM, Munich, Germany) at 105 °C for 24 h, and reweighted (m_f_) [2]. *SW* was calculated according to Equation (5):

(5) $$SW\;(\%)=\left(\frac{m_{0}-m_{f}}{m_{0}}\right)\times 100\%$$

#### 2.7.3. Water Vapor Permeability (*WVP*)

The method reported by Condés et al. [30] was used for *WVP* analysis. Each film sample was sealed over a permeation cell containing silica gel (~0% RH), with a circular opening of 0.00317 m^2^, which was stored in a desiccator at 25 °C. To maintain a 100% RH gradient across the film sample, distilled water was placed inside the desiccator. The water vapor transport was determined from the weight gain of the permeation cell when steady state conditions were reached. Seven weight measurements were made over 7 h, which were plotted as a function of time. The *WVP* was determined using Equation (6).

(6) $$WVP=\left(\frac{W}{t}\right)\times \left(\frac{x}{A\;∆P}\right)$$

where *WVP* is water vapor permeability, W/t is the angular coefficient of the linear regression (g/s), x is the film thickness (m), A is the permeation area (0.0032 m^2^), and ΔP is the partial vapor pressure difference between the dry atmosphere and water (2642 Pa at 25 °C). The results were expressed as g⋅m^−1^⋅s^−1^⋅Pa^−1^, and three replicates per film were measured.

#### 2.7.4. Optical Properties

Film color parameters were evaluated in the CIELAB color space using a spectrocolorimeter (CM5, Konica Minolta, Denver, CO, USA) according to the method reported by Pérez-Córdoba et al. [1]. The spectrocolorimeter was previously calibrated with a standard white tile (*L*_0_* = 96:85; *a*_0_* = 0.02; *b*_0_* = 2.39). The color parameters L* (lightness), a* (redness), and b* (yellowness) were measured at three different random positions over the film surface, and the total color difference (ΔE*) was calculated using Equation (7).

(7) $${\Delta E}^{*}=\sqrt{{\left(L^{*}-L_{0}^{*}\right)}^{2}+{\left(a^{*}-a_{0}^{*}\right)}^{2}+{\left(b^{*}-b_{0}^{*}\right)}^{2}}$$

The yellowness index (*YI*) and whiteness index (*WI*) were calculated using Equations (8) and (9).

(8) $$YI=142.86\frac{b^{*}}{L^{*}}\;$$

(9) $$WI=\sqrt{100-{\left(100-L^{*}\right)}^{2}+{\left(a^{*}\right)}^{2}+{\left(b^{*}\right)}^{2}}\;$$

The opacity of the films was measured using a light transmission barrier assay according to the methodology described by Condés et al. [30]. Briefly, three film samples were cut into rectangular pieces and placed into the cell of the UV-Vis spectrophotometer (Genesys 10S UV-Vis, Thermo Scientific, Madison, WI, USA). The spectrum of each film was measured in transmittance mode (200–800 nm). The opacity was calculated using Equation (10), proposed by Qian et al. [31], and expressed as mm^−1^.

(10) $$Opacity\;\left({mm}^{-1}\right)={Abs}_{\left(500\;nm\right)}/film\;thickness\;\left(mm\right)$$

#### 2.7.5. Mechanical Properties

The tensile strength (*TS*) and elongation at break (*EB*) of the PYS:TG and PYS-OSA:TG films were determined according to the methodology reported by Pérez-Córdoba et al. [1]. The tensile test was performed using a texture analyzer machine (5984, Instron, Norwood, MA, USA) with a tensile grip probe. The films were cut into strips (7 cm × 1.5 cm) and tested with a grip separation of 50 mm and a speed rate of 1 mm/s till breaking. For *TS* and *EB* measurements, at least 10 strips from each film sample were performed, and Bluehill 3 software (Bluehill^®^, Instron, Norwood, MA, USA) was used for collecting data.

#### 2.7.6. Disintegrability Test

An expanded polystyrene box (~5 L) was filled with compost provided by the Planta de Compostaje from Universidad Nacional Agraria La Molina (Lima, Peru), which was used to test the disintegrability of the films, according to the method of Pérez-Córdoba et al. [2]. The films were cut as disks (2 cm in diameter) and dried in a forced-air oven (Venticell 55, MMM, Munich, Germany) at 105 °C for 24 h until they reached a constant weight. Once dried, samples were entirely buried in the wet compost (10 cm in depth) and left for 5 days under aerobic conditions. The box was maintained at 25 °C with the daily addition of water to avoid evaporation, and was held at ~50% relative humidity. The samples were taken out carefully from the wet compost for 5 days and gently washed with distilled water to remove the compost adhered to the individual meshes and the surface of the films. Then, the samples were dried at 105 °C for 24 h and reweighed. The percentage of disintegration was calculated using Equation (11) proposed by Goswaim and Maiti [32].

(11) $$D\left(\%\right)=\left[\left(W_{0}-W_{t}\right)/W_{0}\right]\times 100$$

where *D* is the disintegrability, *W*_0_ is the initial weight of the dry film, and *W_t_* is the weight of the degraded film at 5 days.

### 2.8. Statistical Analysis

All parametric data (except for the SEM) were analyzed using STATGRAPHICS Centurion 18 software (StatPoint^®^ Inc., Warrenton, VA, USA). The results were expressed as the mean ± standard deviation. One-way analysis of variance (ANOVA) and the LSD Multiple Range Test were utilized to determine the significant differences among treatments. Differences were considered significant at *p* ≤ 0.05 in all cases.

## 3. Results and Discussion

### 3.1. Proximate Analysis of Peruvian Purple Yam Tubers (PYTs) and Their Starch (PYS)

The proximate composition analysis of PYTs and PYS is presented in Table 1. Regarding PYTs, the moisture content was consistent with those reported by Pérez et al. [12] (75.28%) and Oliveira et al. [33] (70%). The protein content of PYTs was consistent with that reported by Oliveira et al. [33] (7.64%) but higher than that reported by Pérez et al. [12] (4.87%); however, Pérez et al. [12] reported a similar content (6.79%) for white yam tubers. The ash, fat, and fiber contents were higher than those reported by Pérez et al. [12] and Oliveira et al. [33]. The phosphorus content of PYTs was higher (0.10%) than that reported by Pérez et al. [12] (0.05%). The differences observed could be attributed to factors such as fruit development, soil type, harvest season, maturity stage, climate, fertility, and post-harvest handling of the tuber.

Regarding PYS, the moisture content was comparable with that reported by Pérez et al. [34] (12.7%) and Rached et al. [35] (11.5%) but was higher than that reported by Pérez et al. [12] (8.29%), Oliveira et al. [33] (8.76%), and da Costa et al. [4] (2.3%). The protein content of PYS was lower than that reported by Sharlina et al. [36] (1.34%), da Costa et al. [4] (0.5%), and Oliveira et al. [33] (0.9%) for different *Dioscorea* species; however, the fat content of PYS was higher than that reported by Rached et al. [35] (0.02%), Pérez et al. [12] (0.08%), da Costa et al. [4] (0.12%), and Oliveira et al. [33] (0.02%). The fiber content was lower than that reported by da Costa et al. [4] (1.6%) and Oliveira et al. [33] (2.14%). However, the ash content obtained in this study was consistent with that reported by Pérez et al. [12] (0.08%), while the phosphorus content (0.013%) was lower than that reported by Pérez et al. [12] (0.07%) and Oliveira et al. [33] (0.024%). This proximate composition analysis shows that PYS is suitable for developing biodegradable material.

### 3.2. Starch Characterization

#### 3.2.1. Apparent Amylose Content and Degree of Substitution (DS)

The environmental factors and genotypes among species produce differences in the amylose content of starch per plant [37]. The apparent amylose content of the PYS was higher (30.69%) than the esterified starch (PYS-OSA) (19.97%) (Table 2); a similar behavior pattern was reported by Martínez et al. [24] and López-Silva et al. [38] for esterified native potatoes and corn starches, respectively. These authors proposed that OSA esterification decreases the apparent amylose content because esterification mainly occurs in amorphous domains (amylose chains). In this study, the apparent amylose content values are consistent with those reported by Martínez et al. [5] (23.4−35.4%) for Andean potato (Solanum tuberosum) starches; in addition, these values were higher than those reported by Jiang et al. [13] (9.9–23.9%) but smaller than those reported by Sharlina et al. [36] (44.47%) for starches from other species of *Dioscorea*. The esterification process reduces amylose content and enhances starch functionality; thus, starch with a low amylose content impacts the mechanical properties of starch-based films.

The US FDA has approved OSA for use in starch esterification for food applications with the requirement that it should be used at a level of 3% (DS~0.02). DS refers to the substitution of OH groups per glucose molecule, and its value for PYS-OSA was consistent with that reported by Zhang et al. [39] (0.0146) for esterified corn starch and US-treated (500 W, 5 min) corn starches and Martínez et al. [24] (0.0098−0.0146) for esterified native potato starches with US-assisted treatment (200 W, 5 min). According to Zhang et al. [39], US-assisted treatment enhances the chemical activity of starch esterification.

#### 3.2.2. SEM and Particle Size Distribution

**Figure 2:**
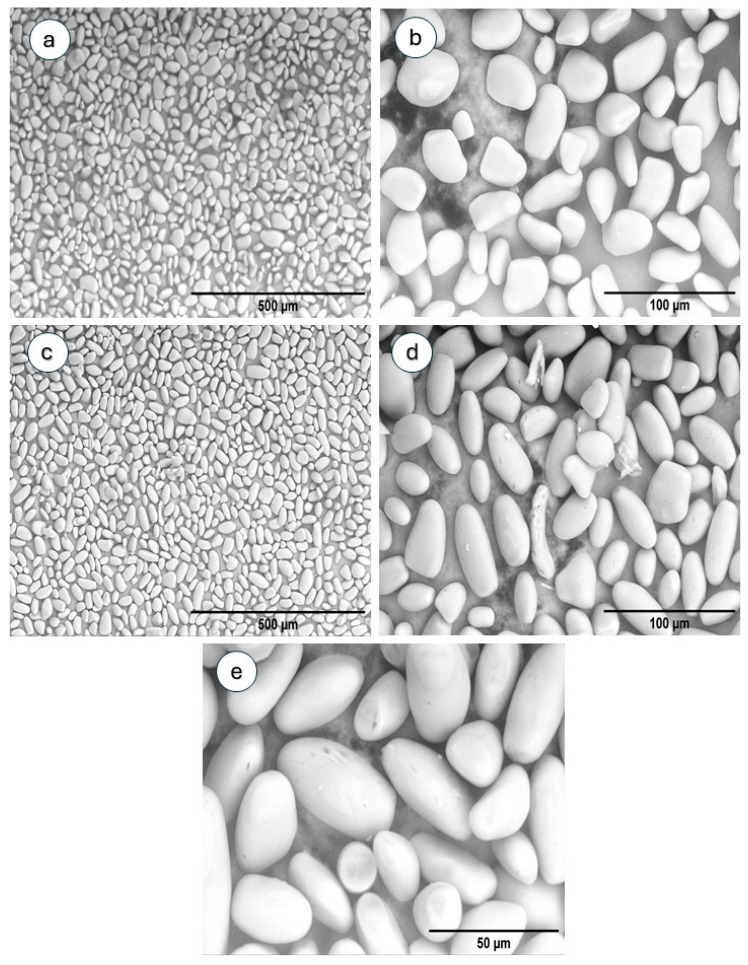
SEM micrographs of PYS (native) and PYS-OSA (esterified) granules: (**a**,**b**) PYS (micrographs of each starch sample were taken at 400× and 1500× magnification) and (**c**–**e**) PYS-OSA (micrographs of each starch sample were taken at 400×, 1500×, and 3000× magnification, respectively).

Emmambux and Taylor [40] stated that the development and size of starch granules are influenced by the plants’ physiology and growing conditions. The micrographs assessed by SEM of PYS and PYS-OSA granules are shown in Figure 2. The PYS granules (Figure 2a,b) showed smooth surfaces without pores, with irregular oval and ellipsoidal shapes. A similar shape was also reported by da Costa et al. [4], Pérez et al. [12], Oliveira et al. [33], and Mao et al. [41] for starch granules of *Dioscorea* species in their works. After OSA esterification, some of the PYS granules were corroded. The surface became rough (Figure 2c,d), and some holes and cracks were observed in the starch particles (Figure 2e) owing to esterification. This may be due to the esterification reaction, which only occurs in the amorphous region of the starch surface, causing light damage on the surface of the granules [24,31,38,42]. However, the rheological test of starch gels showed that morphological changes in starch granules may not affect the starch/tara gum film-forming dispersion (FFD).

**Figure 3:**
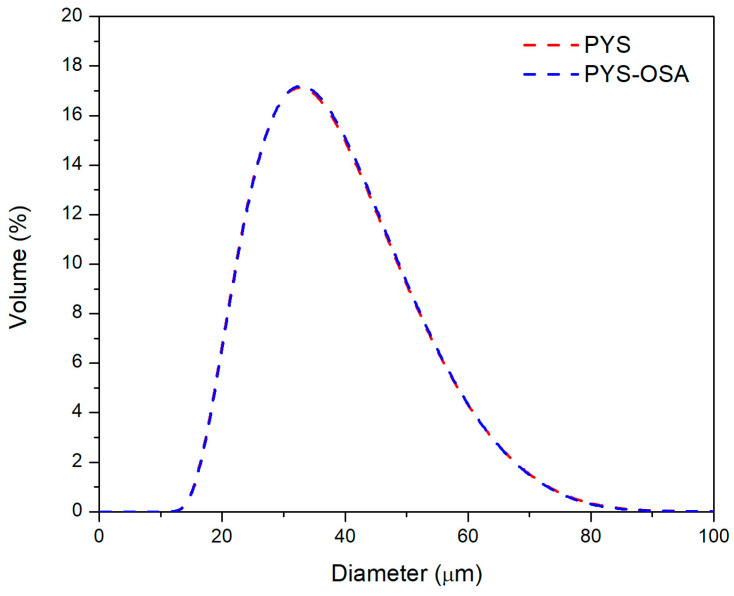
Particle size distribution for PYS and PYS-OSA.

The particle size distributions of both PYS and PYS-OSA granules were similar (Figure 3) and exhibited a unimodal size distribution. The particle size diameters of the granules ranged from 11.94 to 92.05 μm, which is close to that of native potato and tunta starches (10–80 μm) reported by Martínez et al. [28]. In this study, the results showed that esterification did not affect particle size [31]. Also, the volume-based mean diameters (*D* [4,3]) of PYS and PYS-OSA are presented in Table 2.

#### 3.2.3. FT-IR Analysis

**Figure 4:**
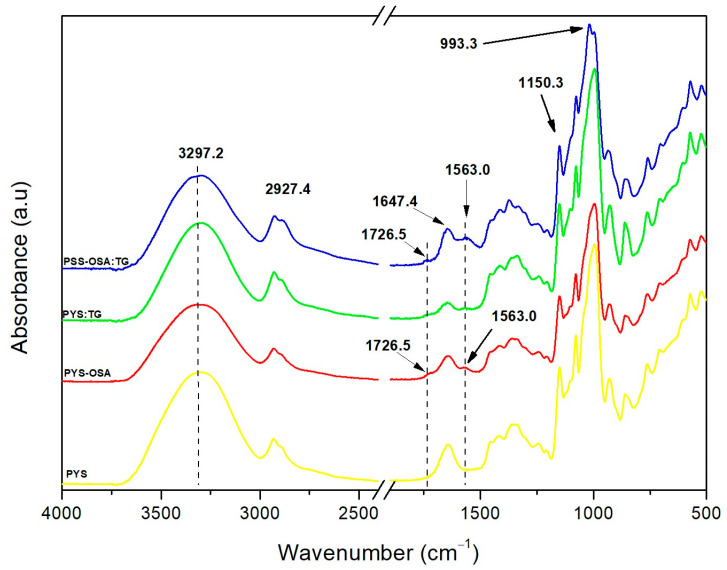
FT-IR spectra for PYS and PYS-OSA and PYS:TG and PYS-OSA:TG films.

The information on chemical groups and their vibrational state, relating to changes in the chemical composition of the materials, is provided by FT-IR analysis [4]. The normalized FT-IR spectra of PYS and PYS-OSA are shown in Figure 4. For both starches, the fingerprint region is located between 900 and 1120 cm^−1^ [4], the polar double bonds region between 1300 and 1800 cm^−1^, the triple links region between 1800 and 2700 cm^−1^, and the polar hydrogen bonds region between 2700 and 3750 cm^−1^ [4]. Nevertheless, due to esterification with OSA, the FT-IR spectrum of PYS-OSA showed two new absorption bands at 1726 and 1563 cm^−1^, which correspond to the ester carbonyl groups and the asymmetric stretching vibration of the carboxylate, respectively. In this line, Martínez et al. [24] and Chakraborty et al. [43] reported similar absorption bands in the esterification of native potato and sorghum starches, respectively, using US-assisted treatment. Due to the chemical changes, OSA starches are useful for preparing biodegradable films that exhibit modifications in their mechanical and thermal properties. Different authors [44,45] have used esterified starches with DSs of (0.013–0.023), which modified the tensile strength of their films.

#### 3.2.4. X-Ray Diffraction and Crystallinity

**Figure 5:**
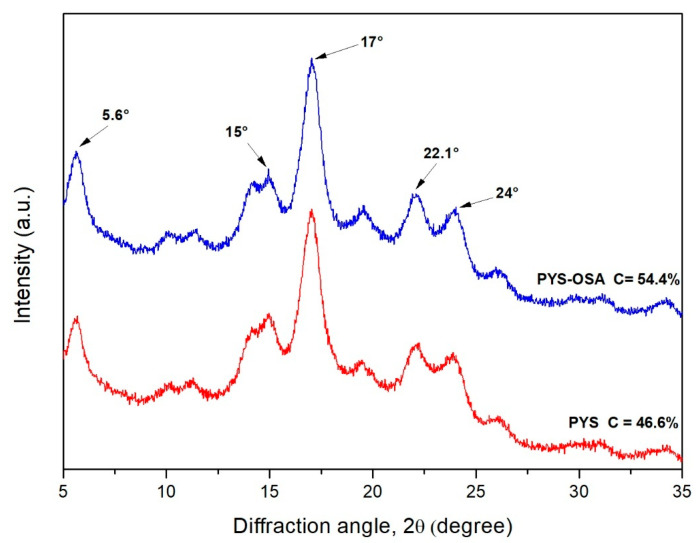
X-ray diffraction pattern for PYS and PYS-OSA.

The relative crystallinity and diffraction patterns of PYS and PYS-OSA are shown in Table 2 and Figure 5, respectively. PYS-OSA showed a higher relative crystallinity value (54.4%) than PYS (46.6%), which could be due to the esterification with OSA taking place mainly in the amorphous region, which is easy to access and more sensitive to esterification than the crystalline region [31]. In this line, Qian et al. [31] reported a similar behavior; thus, the relative crystallinity of the OSA yam starch (41.15%) was slightly higher than that of the native one (39.27%). Both starches display a similar diffraction pattern, with five characteristic peaks at 2θ 5.6°, 15°, 17°, 22°, and 24°. These peaks are characteristic of the B-type crystalline structure, typical in tuber starches, where glucose helices are packed slightly densely, leaving room for water molecules between the branches [46]. The diffraction pattern is related to the chain-length distribution of amylopectin. The X-ray diffraction pattern for PYS-OSA was consistent with those reported by Qian et al. [31] and Martínez et al. [24] for esterified Chinese yam and esterified Andean potato starches, respectively. A higher starch crystallinity is generally associated with improved mechanical properties of films, such as increased tensile strength (*TS*). In this study, modified starch-based films showed an increase in *TS*.

#### 3.2.5. Thermal Properties

The gelatinization temperature and enthalpy of PYS-OSA decreased compared to those of PYS (the native counterpart) (Table 2), which could be due to esterification mainly affecting the amorphous region [24,31]; this is consistent with the results of the XRD analysis. The introduced OS groups in the amorphous zone destabilize the organized structure of the PYS granules; this factor decreased the gelatinization characteristics of PYS-OSA. In addition, the inserted OS groups weaken the interactions between amylose and amylopectin, allowing starch-esterified granules to swell and melt at lower temperatures. Zhang et al. [39] and Martínez et al. [24] reported that Δ*H* decreased in esterified and ultrasound-treated corn and Andean potato starches compared to their native counterparts; therefore, the Δ*H* value of PYS-OSA could be due to these processes. There were statistically significant differences (*p* ≤ 0.05) for *T_o_*, *T_p_*, *T_c_*, and Δ*H* among PYS and PYS-OSA. Despite the decrease in thermal properties, the crystalline zones of amylopectin were maintained, which was confirmed by XRD and FTIR analysis.

#### 3.2.6. Rheological Characteristics

Rotational Tests

**Figure 6:**
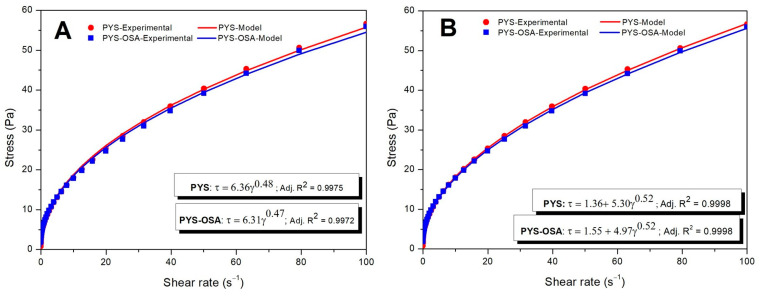
Power law (**A**) and Herschel–Bulkley (**B**) models describing the flow behavior for PYS and PYS-OSA gels at 25 °C.

PYS and PYS-OSA pastes showed shear-thinning behavior (Figure 6). The entangled networks of amylose and amylopectin could be due to the disruption of starch granules under shear. As the shear rate increased, more bonds broke down, which led to a greater molecular alignment that caused a decrease in apparent viscosity [42]; however, US pretreatment did not affect the flow behavior of the PYS-OSA compared to PYS. For both starch pastes, the experimental parameters were well fitted to power law and Herschel–Bulkley models with a coefficient of determination (R^2^) greater than 0.9 (Table 3). In the power law model, the consistency index and flow index of the PYS paste were greater than those of the PYS-OSA; Chakraborty et al. [43] observed a similar trend in these parameters for esterified sorghum starch with ultrasound-assisted treatment. The *σ*_0_ value represents the minimum effort required to make the starch paste flow and indicates the strength level of the starch network. This value for PYS paste (1.36 Pa) was lower than that for PYS-OSA (1.55 Pa) (*p* ≤ 0.05); this difference could be related to amylose content [28]. The PYS-OSA showed a slight decrease in k value (4.97 Pa·s^n^), but the n value (0.52) was the same for both starches. During the film preparation, the shear thinning behavior of the starch gels led to their viscosities decreasing with stirring, which might have enhanced the homogeneity in FFD.

Oscillatory Tests

**Figure 7:**
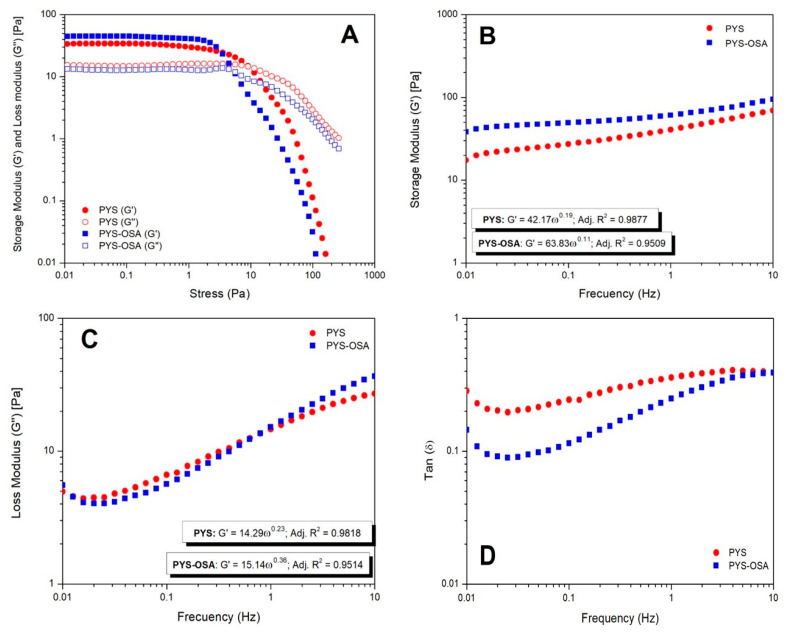
Amplitude sweep analysis (**A**), *G*′ as a function of frequency (**B**), *G*″ as a function of frequency (**C**), and *tan δ* as a function of frequency (**D**) for PYS and PYS-OSA gels at 25 °C.

The amplitude sweep determines the range of linear viscoelasticity (LVE) that represents the threshold where the storage and loss modulus (viscoelastic properties) stay stable despite the increasing stress. This region indicates critical insights into the structure of starch gels under shear conditions [43]. The results show that 1 Pa stress falls within the LVE region of the PYS and PYS-OSA gels. The dynamic mechanical spectra of PYS and PYS-OSA gels show a predominance of elastic over viscous character (*G*′ > *G*″) (Figure 7A), i.e., gel-like behavior, which could influence the FFD to obtain a higher *TS* in films.

Regarding the frequency sweep test, no crossover point was observed between *G*′ and *G*″, as *G*′ consistently remained higher than *G*″, indicating that PYS and PYS-OSA gels exhibited predominantly solid-like behavior (Figure 7B,C). The loss factor could characterize differences in gel strength and viscoelastic behavior (*tan δ*). In both starch gels, *tan δ* was less than 0.4 (Figure 7D), which indicates that PYS and PYS-OSA gels exhibited more elastic behavior [43]. The *G*′ and *G*″ values of PYS-OSA were slightly higher than those of the native form (PYS). This pattern could be due to US pretreatment disrupting the starch structure granules, which caused starch molecules to unfold and enhance the cross-linking of inter- and intramolecular hydrogen bonds in starch granules. Then, the starch granules rearranged internally, reaching a more orderly and stable internal structure; thus, the US pretreatment effect increased the elasticity of the PYS-OSA paste [47]. This finding was contrary to that of Chakraborty et al. [43], who reported that an extreme US process increases starch fragmentation, which could reduce viscoelastic properties.

### 3.3. Film Characterization

#### 3.3.1. Film Appearance and Thickness

The PYS:TG and PYS-OSA:TG films were slightly opaque without bubbles, scratches, or phase separation, and had a similar good general appearance. This appearance can be verified by the opacity values, which did not show a significant difference (Table 3). The thicknesses of the films were maintained due to there being the same mass ratio of FFD in the Petri plate area which was added [1]. These values were approximately equal to those reported by Niu et al. [48] (0.071 mm) for a potato starch/gelatin film (Table 3).

#### 3.3.2. Moisture Content (*MC*), Solubility in Water (*SW*), and Water Vapor Permeability (WVP)

Some physicochemical properties of the PYS:TG and PYS-OSA:TG films, in which water plays an important role, are summarized in Table 3. The PYS-OSA:TG films exhibited the highest *MC* (17.14%) owing to the amphiphilic character of PYS-OSA caused by the introduced OS groups in the starch structure. The PYS-OSA-based films could induce an increase in *MC* greater than that of the PYS:TG films. These results were lower than those reported by Saberi et al. [49] (20%) for an edible film based on pea starch and guar gum; nevertheless, they were approximately equal to those reported by López et al. [50] (10.61−14.19 g water/100 g film) for esterified corn starch films and by Pérez-Córdoba et al. [1] (12.6−13.2%) for nanocomposite tunta starch/tara gum films. Additionally, Shanbhag et al. [51] reported that films containing corn starch blends showed *MC* levels of 6−16%, which are suitable for food packaging. However, *MC* could affect film stability due to the hydrophilic nature of starch; then, it would be important to carry out shelf-life studies to ensure the preservation of the mechanical and stability properties of films.

Regarding the *SW*, PYS-OSA:TG films showed higher *SW* values than PYS:TG ones (Table 3), owing to glycerol (plasticizer), which increases *SW* because of its character of hydrophilicity [50,51]. Nevertheless, both films showed lower *SW* than the values reported by Pérez-Córdoba et al. [1] (28.5−30.9%) for nanocomposite tunta starch/tara gum films. The densities of the PYS:TG and PYS-OSA:TG films are shown in Table 3. The densities of PYS:TG films were greater than those of PYS-OSA:TG ones because of PYS in the films, making the structure more compact. It could be that a higher density of films results in lower *SW* and *WVP*.

*WVP* is a critical property in films developed from hydrophilic materials [48]. Both PYS:TG and PYS-OSA:TG films showed *WVP* values (Table 3) in the same order of magnitude as those reported by da Costa et al. [4] for purple yam starch/chitosan films (1.4 × 10^−10^ g⋅m^−1^⋅s^−1^⋅Pa^−1^) and Niu et al. [48] for potato starch/gelatin films (1.5 × 10^−10^ g⋅m^−1^⋅s^−1^⋅Pa^−1^); nevertheless, Pérez-Córdoba et al. [1] reported higher values (5.9−6.6 × 10^−10^ g⋅m^−1^⋅s^−1^⋅Pa^−1^) for nanocomposite tunta starch/tara gum films. The *WVP* values obtained in this study may be attributed to the formation of intermolecular hydrogen bonds between glycerol and starch with the matrix, resulting in strong interfacial adhesion [48].

#### 3.3.3. Optical Properties

Table 3 displays the color parameters and opacity values measured for the PYS:TG and PYS-OSA:TG films. PYS esterification with OSA slightly increased the lightness of the PYS-OSA:TG films; the values of these results were higher than those reported by Pajak et al. [52], who reported a reduction in L* values in octenyl succinylated potato-starch-based films enriched with extracts. Also, the PYS-OSA:TG films showed a significant difference (*p* ≤ 0.05) compared to PYS:TG films in terms of the color difference (ΔE*), which may be due to the esterification of PYS, causing the insertion of OS groups in the starch structure. In this study, the ΔE* values for PYS:TG and PYS-OSA:TG films were higher and lower than those reported by Pérez-Córdoba et al. [1] (0.76). A similar pattern of behavior was shown for the yellowness index (*YI*); the value for PYS:TG films was higher than that for PYS-OSA:TG ones, and both were lower than that reported by Pérez-Córdoba et al. [1] (4.49). Regarding the whiteness index (*WI*), both films exhibited similar behavior in terms of L* values.

The light transmittance (%) in the visible region (400–800 nm) of the PYS:TG films reached the maximum value, remaining constant at approximately 80% and then exhibiting a lower opacity value than PYS-OSA:TG films, as shown in Table 3. The film transparency is indirectly measured by opacity [53]; thus, the film’s high opacity value restricts the packed product’s visibility from outside of the package [54]. The slightly higher opacity value for PYS:TG films than for PYS-OSA:TG ones (Table 3) could be due to the compaction of high polymeric chains between PYS-OSA and TG, which obstruct the transmission of light across the film [52]. Additionally, opacity could be increased due to the phase separation process, in which TG interacts with amylose and amylopectin through non-covalent hydrogen bonds and forms different network structures [53,55]. In addition, transparency and opacity are critical properties when designing films because they impact the film’s functionality in protecting food from light that induces some spoilage reactions while maintaining consumer interest [56].

#### 3.3.4. Mechanical Properties

**Figure 8:**
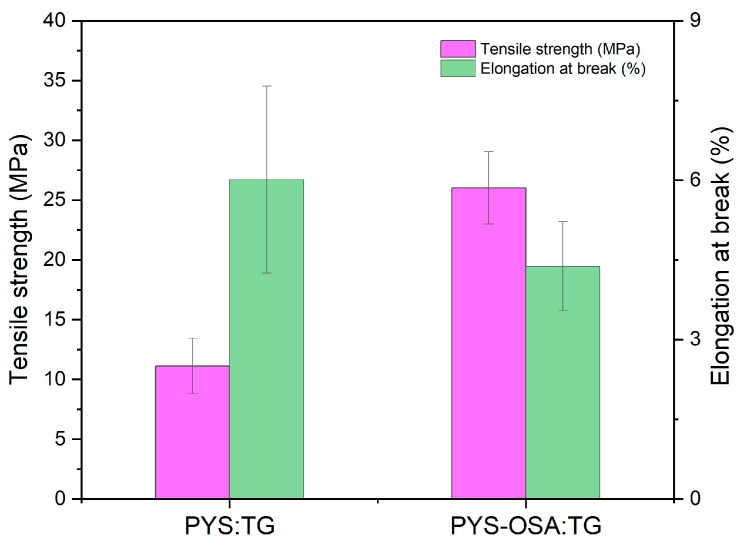
Tensile strength (*TS*) and elongation at break (*EB*) of PYS:TG and PYS-OSA:TG films. Bars indicate standard deviation for three replicates.

Figure 8 shows the mechanical properties of PYS:TG and PYS-OSA:TG films. It is known that tensile strength (*TS*) measures the maximum strength of the films before breaking. PYS-OSA:TG films containing modified starch with TG showed the highest *TS* value (26.91 MPa), suggesting higher strength than PYS:TG ones made with native starch (11.12 MPa). According to Mukherjee et al. [15], films made with TG have certain drawbacks, such as low tensile strength; however, the PYS-OSA:TG matrix caused a significant (*p* ≤ 0.05) increase in the tensile strength of the films, owing to TG helping in the production of strong and flexible films [15], so they could generate additional bonds that strengthened the structure of the material [57]. Regarding strain value, this indicates the ability of films to elongate before breaking (elongation at break, *EB*). PYS:TG films show a higher deformation value (6.01%), indicating that these are more elastic or flexible than PYS-OSA:TG (4.38%) ones, i.e., PYS:TG films show a lower deformation, suggesting that these films show less elasticity and more stiffness. Furthermore, Karnwal et al. [56] mentioned that the amylose–amylopectin ratio influences film properties, i.e., high-amylose starches produce rigid films with increased *TS* but reduced *EB* due to enhanced crystallinity.

#### 3.3.5. FT-IR Analysis of Films

Figure 4 depicts the FTIR spectra and chemical structure of the developed PYS:TG and PYS-OSA:TG films, which show peaks with no differences compared with PYS and PYS-OSA. The absorption broadband at 3297 cm^−1^ in films corresponds to the stretching vibration of the hydroxyl group connected with the inter- and intramolecular bonds of the –OH group of nearby starch molecules, displaying the formation of a hydrogen bond between the constituents in each film [51], while the absorption band at 2927 cm^−1^ corresponds to the stretching vibrations of C–H stretching [4] and the C–H_2_ bonds of glycerol [51]. As was expected, due to esterification, the PYS-OSA:TG films showed absorption bands at 1726 and 1563 cm^−1^, which corresponded to the ester carbonyl groups and the asymmetric stretching vibration of the carboxylate, respectively.

#### 3.3.6. Disintegrability Test

**Figure 9:**
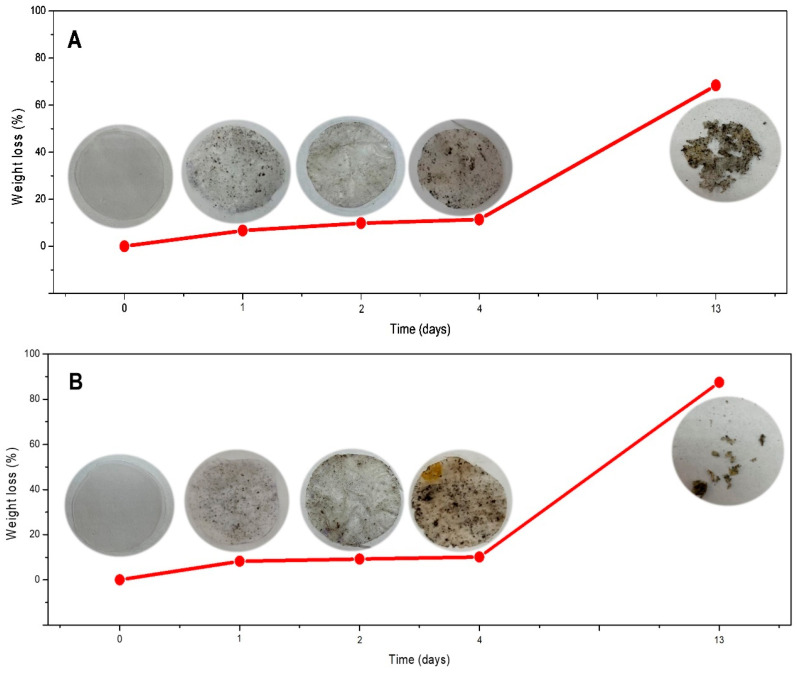
Disintegrability test results of (**A**) PYS:TG and (**B**) PYS-OSA:TG films in soil.

Film disintegration occurs owing to the action of enzymes and involves living soil microorganisms. Enzymes promote molecular degradation and can occur under both aerobic and anaerobic conditions, resulting in partial or complete degradation of the environment [58]. He et al. [47] mentioned that the films have a hydrophilic nature, whereby water enters the polymer chains, weakening them and allowing them to be hydrolyzed by soil microorganisms. The compost soil used for the disintegrability test was reported by Pérez-Córdoba et al. [2]. In this study, PYS:TG and PYS-OSA:TG films exhibited mass losses (disintegrability) of 70% and 90%, respectively, during the 13 days of testing under controlled composting conditions, indicating a positive biodegradation process (Figure 9). This is due to the relationship between the available moisture and the enzymes of the soil compost microorganisms on the films [2]. The rapid disintegration of PYS-OSA:TG films could be due to their lower intermolecular interactions between PYS-OSA and TG, making them more susceptible to degradation by microorganisms [58]; as the modification of PYS with OSA altered the native starch structure [24], the low molecular order of PYS-OSA could cause a greater mass loss of PYS-OSA:TG films. Despite starch offering a sustainable alternative to plastic, it is essential to assess the environmental footprint of starch films across the production, usage, and disposal stages of industrial production. The study of viability can be conducted by a Life Cycle Assessment (LCA) of the early design stages to reduce environmental impact while maximizing resource efficiency. In addition, it would be important to investigate the release of specific components that could be ecotoxic.

## 4. Conclusions

PYS and PYS-OSA showed good physical properties in order to be used for the development of starch-based films which could contribute to mitigating environmental pollution. PYS and PYS-OSA were blended with TG and glycerol to obtain films by the casting method (PYS:TG and PYS-OSA:TG films) and characterized through several techniques. PYS-OSA films showed higher solubility in water, optical properties, and tensile strength than PYS films. Nevertheless, PYS-OSA:TG films exhibited mass losses of 90% under controlled composting conditions. These characteristics demonstrated that PYS and PYS-OSA films could be a feasible packaging for food products and could improve shelf-life. Starch from non-conventional crops, such as yam, could be an important material in the development of food packaging. Nevertheless, it is important to increase the production of these crops and carry out more research work to enhance the packages’ properties.

In the future, when petroleum-derived plastics are no longer used, starch will be one of the most important materials for the biodegradable packaging industry. Therefore, it would be of socioeconomic importance for all tropical countries producing these underutilized crops to increase its agro-industrial potential and market value.

## Figures and Tables

**Table 1:** Chemical characteristics of purple yam tubers (PYTs) and their starch (PYS).

Component	PYTs	PYS
Moisture content (%)	76.42 ± 0.16	13.53 ± 0.02
Total protein (%, db)	6.87 ± 0.29	0.31 ± 0.10
Fat (%, db)	1.02 ± 0.23	1.47 ± 0.03
Fiber (%, db)	2.71 ± 0.14	0.13 ± 0.02
Ash (%, db)	3.27 ± 0.08	0.06 ± 0.00
Phosphorus (mg/100 g, db)	94.72 ± 0.72	13.38 ± 0.04

Values expressed as mean ± standard deviation (n = 3).

**Table 2:** Apparent amylose content, color parameters, particle size, crystallinity, and gelatinization properties of PYS and PYS-OSA.

Component	PYS	PYS-OSA
Apparent amylose content (%)	30.69 ± 0.72 ^b^	19.97 ± 0.16 ^a^
Degree of substitution	--	0.023
Color parameters		
L*	93.59 ± 0.05 ^a^	95.96 ± 0.39 ^b^
a*	2.29 ± 0.16 ^b^	0.08 ± 0.02 ^a^
b*	1.08 ± 0.06 ^a^	1.32 ± 0.14 ^a^
*W* (%)	93.11 ± 0.02 ^a^	95.61 ± 0.25 ^b^
Particle size distribution		
D [4,3] (μm)	34.5	34.9
Crystallinity (%)	46.6	54.4
Differential scanning calorimetry		
*T_o_* (°C)	71.75 ± 0.09 ^b^	68.30 ± 0.59 ^a^
*T_p_* (°C)	75.83 ± 0.08 ^b^	73.27 ± 0.06 ^a^
*T_c_* (°C)	81.57 ± 0.16 ^b^	78.81 ± 0.18 ^a^
Δ*H* (J/g)	14.27 ± 0.11 ^a^	13.18 ± 1.43 ^a^

Values expressed as mean ± standard deviation (n = 3). Means in the same row followed by different letters are significantly different (*p* ≤ 0.05). *T_o_*, onset temperature; *T_p_*, peak temperature; *T_c_*, conclusion temperature; Δ*H*, gelatinization enthalpy (on dry basis).

**Table 3:** Characterization of the PYS:TG and PYS-OSA:TG films.

Characteristic	PYS:TG	PYS-OSA:TG
Moisture content (%)	11.37 ± 1.67 ^a^	17.14 ± 0.87 ^b^
Thickness (mm)	0.076 ± 0.004 ^a^	0.074 ± 0.002 ^a^
Solubility in water (%)	0.25 ± 0.06 ^b^	0.42 ± 0.09 ^a^
*WVP* × 10^−10^ (g·m^−1^ s^−1^ Pa^−1^)	1.10 ± 0.26 ^a^	1.33 ± 0.22 ^b^
Density (g/cm^3^)	2.02 ± 0.02 ^a^	1.93 ± 0.11 ^b^
Optical properties		
L*	96.65 ± 0.26 ^a^	97.09 ± 0.05 ^b^
ΔE*	2.74 ± 0.24 ^b^	2.17 ± 0.05 ^a^
*YI*	0.29 ± 0.03 ^b^	−0.05 ± 0.00 ^a^
*WI*	96.50 ± 0.26 ^a^	97.06 ± 0.00 ^b^
Opacity (mm^−1^)	2.42 ± 0.26 ^a^	2.76 ± 0.02 ^a^

Values expressed as mean ± standard deviation (n = 3). Means in the same row followed by different letters are significantly different (*p* ≤ 0.05).

## Data Availability

The original contributions presented in this study are included in the article; further inquiries can be directed to the corresponding authors.
